# MR Imaging of Hemosiderin Deposition in the Ankle Joints of Patients with Haemophilia: The Contribution of a Multi-Echo Gradient-Echo Sequence—Correlation with Osteochondral Changes and the Number and Chronicity of Joint Bleeds

**DOI:** 10.3390/life14091112

**Published:** 2024-09-04

**Authors:** Olympia Papakonstantinou, Efstratios Karavasilis, Epaminondas Martzoukos, Georgios Velonakis, Nikolaos Kelekis, Helen Pergantou

**Affiliations:** 1 2nd Department of Radiology, Attikon General University Hospital, School of Medicine, National and Kapodistrian University of Athens, 12462 Athens, Greece; sogofianol@gmail.com (O.P.); stratoskaravasilis@yahoo.gr (E.K.); nonnontas@gmail.com (E.M.); giorvelonakis@gmail.com (G.V.); kelnik@med.uoa.gr (N.K.); 2 Research Unit of Radiology and Medical Imaging, 2nd Department of Radiology, Attikon General University Hospital, School of Medicine, National and Kapodistrian University of Athens, 11527 Athens, Greece; 3 Haemostasis and Thrombosis Unit, Haemophilia Centre, Aghia Sophia Children’s Hospital, 11527 Athens, Greece

**Keywords:** haemophilia, haemophilic arthropathy, MRI, hemosiderin deposition, joint bleeds, hemarthrosis

## Abstract

We aim (a) to introduce an easy-to-perform multi-echo gradient-echo sequence (mGRE) for the detection of hemosiderin deposition in the ankle joints of boys with haemophilia (b) to explore the associations between the presence and severity of hemosiderin deposition and the other components of haemophilic arthropathy, the clinical score, and the number and chronicity of joint bleeds. An MRI of 41 ankle joints of 21 haemophilic boys was performed on a 3 T MRI system using an mGRE sequence in addition to the conventional protocol. Conventional MRI and mGRE were separately and independently assessed by three readers, namely, two musculoskeletal radiologists and a general radiologist for joint hemosiderin. We set as a reference the consensus reading of the two musculoskeletal radiologists, who also evaluated the presence of synovial thickening, effusion, and osteochondral changes. Excellent inter-reader agreement was obtained using the mGRE sequence compared to the conventional protocol (ICC: 0.95–0.97 versus 0.48–0.89), with superior sensitivity (90–95% versus 50–85%), specificity (95.2–100% versus 76.2–95.2%), and positive (95–100% versus 71–94.4%) and negative predictive value (91.3–95.5% versus 87–63%). Hemosiderin deposition was associated with osteochondral changes, synovial thickening, clinical score, and the total number of ankle bleeds, while it was inversely related with the time elapsed between the last joint bleed and MRI. (*p* < 0.05). The application of an mGRE sequence significantly improved hemosiderin detection, even when performed by the less experienced reader. Joint hemosiderin deposition was associated with the other components of haemophilic arthropathy and was mostly apparent in recent joint bleeds.

## 1. Introduction

Haemophilia A is an X-linked recessive disorder of blood coagulation occurring due to a congenital deficiency of Factor VIII (FVIII) [1]. Haemophilic arthropathy (HA), due to repeated haemarthroses, mostly involves ankles, knees and elbows and constitutes the major cause of morbidity in patients with haemophilia (PWH). Haemophilia can be categorized into mild, moderate, and severe forms based on the amount of coagulation factors present. Each exhibits a different propensity for recurrent joint bleedings, which significantly impacts the management and treatment strategies used for affected individuals [1,2,3]. Prophylactic treatment with FVIII concentrates significantly reduces the incidence of joint bleeds (JBs), with dramatic improvement in the quality of life of PWH. The early detection of HA is of paramount importance for the prompt treatment of PWH and the evaluation of prophylactic regimens and schemes [4,5,6,7].

MR imaging has been established as the reference imaging method for the detection of even subclinical bleeds [6,8,9,10,11,12] and the assessment of the severity of HA [8,9,13,14,15] over the whole spectrum of joints [9,15,16,17,18,19]. Most recently, the predictive value of early MRI findings for future joint bleeds and the development of haemophilic arthropathy has been emphasized [17,18,19]. However, to the best of our knowledge, an MRI protocol for the evaluation of HA has not been standardized and published series employ various MRI techniques. Although it is well known that GRE MRI techniques are the most sensitive in the detection of hemosiderin in tissues [9,14,20], comparison between GRE and conventional MRI protocol has not been adequately addressed, whereas the interpretation of the MRI studies has been performed by radiologists with focused experience on imaging of HA, who are often developers of quantitative MRI scales for the evaluation of HA [21]. During recent years, ultrasound has emerged as a more economic and easily accessible method for the evaluation of JBs and HA, with the capability to be employed as a first-line point-of-care screening method [16,22,23,24]; however, its global efficacy is hampered by equivocal capabilities in the identification of hemosiderin deposition and an inability to visualize deep-seated structures within the joint and osseous structures beyond the bone cortex [24,25,26,27].

In our institution, we employ a multi-echo gradient-echo sequence (mGRE) sequence to identify joint hemosiderin as we have observed that the gradual decrease in the signal of the mGRE sequence enhances the sensitivity and confidence of readers in the detection of even mild joint hemosiderin deposition; moreover, it is an easily applicable technique in everyday clinical practice. To the best of our knowledge, the application of mGRE sequences in the evaluation of hemosiderin deposition in HA has not been reported.

The purpose of our study is twofold: first, we aim to evaluate the feasibility of using mGRE sequence to detect the presence of intraarticular hemosiderin in the ankle joints in young PWH. Second, we seek to explore the relation between the degree of hemosiderin deposition within the joint (JHD) and the other components of HA, i.e., synovial thickening, effusion, osteochondral changes, the clinical score, and the number and chronicity of joint bleeds. This is because the relation between the presence of hemosiderin and the chronicity of joint bleeds has not been fully explored [10,17,28,29]. We focused on ankle joints as these are the most common location of bleeding episodes and of subclinical bleeds in young PWH; moreover, the involvement of the ankle joint affects significantly gait and posture [30,31].

## 2. Methods

### 2.1. Patients

We included magnetic resonance imaging studies of 41 ankle joints of 21 consecutive PWH (age range 9–17 years, mean: 14.7 ± 2.7 years) and 10 ankle joints of 5 healthy controls (HC) (age range:10–18 years, mean: 13.2 ± 3.42 years) in this prospective cohort study, performed within a 31-month period (September 2019 to April 2022). The patients were randomly selected from 105 children with haemophilia A, followed at our Haemophilia Comprehensive Care Centre (HCCC). The inclusion criteria for all subjects included an age > 9 years old, the absence of a history of recent trauma in a period of less than six months from the MRI study, and the absence of congenital malformations of the foot and ankle or rheumatologic disease. The severity of the disease was not considered when including patients. The age limit was set at 9 years, as younger children do not present with substantial complaints of arthropathy in our Centre due to the implementation of primary prophylaxis in children with severe haemophilia before the age of 2 years or after the first joint bleed. Moreover, children older than 9 years do not need general anesthesia (sedation) to undergo MRI, facilitating the conduct of our study. All patients were negative for FVIII inhibitors towards FVIII concentrates. Informed consent was obtained from the parents of all subjects and we obtained assent from subjects older than 12 years. This study obtained approval from the ethics committee of our hospital. The parents of the five healthy controls were staff members of the HCCC; both children and their parents consented to participation in the study since no invasive procedures were performed. Sixteen boys had severe, two moderate, and three mild haemophilia A. All children with severe haemophilia and one with moderate were under prophylaxis, early (*n* = 8) or late (*n* = 9), while the three boys with mild haemophilia A and one with moderate used on-demand treatment, all being treated by recombinant FVIII concentrates. Eight patients with severe haemophilia were placed on early prophylaxis, initiated immediately after the first joint bleed, at a mean age of 15 months, whereas the remaining patients, all with milder clinical phenotypes, started prophylaxis later, at a mean age of 4 years. The number of lifetime joint bleeds and the time elapsed between the last bleed and the MRI study were recorded from the patients’ files. The Haemophilia Joint Health Score (HJHS) 2.1 was applied for all patients between 1 and 3 weeks before MRI for each individual joint; the ankle joints of our study represented a wide spectrum of clinical severity in terms of HA, ranging from normal to severe. The HJHS 2.1 is a validated outcome tool for children aged > 5 years old and is used for the physical assessment of the elbows, the knees, and the ankles. Higher scores indicate poorer joint status, with a total score ranging from 0 to 124 [32].

### 2.2. MRI Technique

MRI studies of the ankle joints of all patients and healthy controls were performed on the same 3 T Achieva TX Philips MRI scanner (Philips, Best, The Netherlands), equipped with an 8-channel ankle coil, using the same imaging protocol for all subjects. Participants were instructed to remain still and foam pads were also positioned to stabilize the joints. None of the MRI scans suffered from motion artifacts. The MRI technique compromised a conventional MRI protocol, including the use of turbo spin-echo (TSE) and short-tau inversion recovery (STIR) sequences on the sagittal plane (T1 TSE, Repetition Time (TR): 590 ms; echo time (TE): 20 ms; Slice Thickness (ST): 2.5 mm; reconstruction matrix: 640 × 640; T2 TSE, TR: 3228 ms; TE: 120 ms; ST: 2.5 mm, reconstruction matrix: 640 × 640; T2 Short-TI Inversion Recovery (STIR), TR: 3947 ms; TE: 30 ms; ST: 2.5 mm; Inversion Recovery (IR), Inversion time: 190 ms; reconstruction matrix: 640 × 640). Subsequently, we applied a sagittal multi-slice and multi-echo GRE sequence with 8 equidistant echoes (TR: 180 ms, TE1: 2.3 ms, ΔTE: 2.3 ms, ST: 2.5 mm, pixel bandwidth: 885 Hz, reconstruction matrix: 512 × 512, 22 slices, number of signal averages: 2, flip angle: 55°). The duration of m-GRE acquisition was 5 min and 8 s.

### 2.3. Imaging Analysis and Interpretation

**Figure 1:**
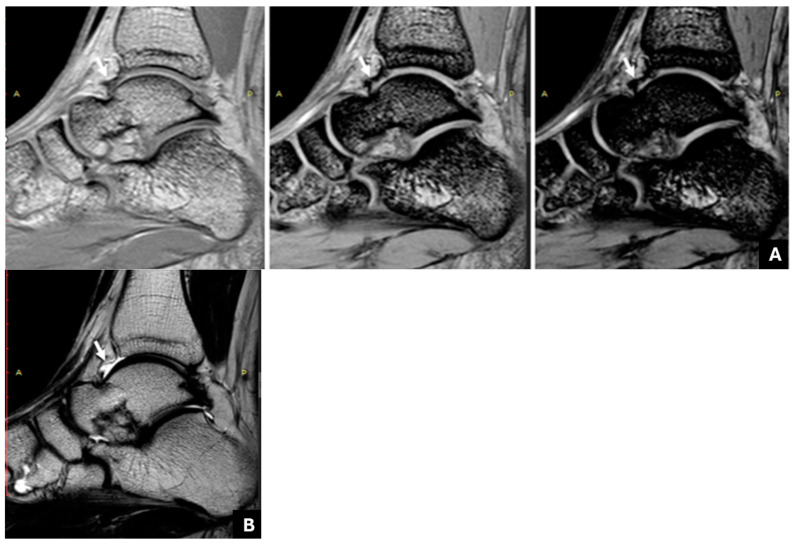
Right ankle joint of a 14-year-old patient with haemophilia A and a history of four joint bleeds. (**A**): The multi-echo GRE (mGRE) sequence (TR: 180 ms, TE1: 2.3 ms, ΔTE: 2.3 ms, flip angle: 55°) shows the progressive signal drop in the synovium at an area of the anterior recess (indicated by the arrow) of the right ankle joint ((**A**) from right to left: 1TE: 2.3, 4TE: 9.2, 6TE: 13.8), suggesting hemosiderin deposition. (**B**): This finding is not shown in the corresponding sagittal T2W TSE image of the same area (arrow).

Using the methodology reported by Akuyz et al., the feasibility of using mGRE sequences to assess JHD was assessed by three independent readers with variable experience in reading joints of PWH [29]: two musculoskeletal radiologists with 18 and 9 years of experience in MSK imaging and 13 and 4 years of experience in the imaging of joints of PWH (readers A and B), and a general radiologist (reader C). Reader C was not specialized in MSK imaging and did not have any focused experience on the imaging of joints with haemophilia, whereas MSK MRI comprised less than 20% of his daily practice over the last 10 years. Before independent readings, all three readers standardized, by consensus, the interpretation of JHD using, first, the conventional protocol and then the mGRE sequence in three cases. Subsequently, the readers reviewed, by consensus, the 10 joints of HC (both conventional protocol and mGRE) and agreed that there were no signs of JHD in any of them. With the mGRE sequence, the healthy controls presented a mild reduction in signal with increasing TE, as expected, but the signal did not reach a signal void and the blooming artifact was not apparent in any image. Then, conventional MR imaging studies (including T1W and T2W TSE and STIR images) of all patients were randomly presented to each one of the three reviewers, who were blind to the patients’ names, clinical history, and initial MRI reports; each reviewer decided independently on the presence or absence of JHD based on previously published criteria [15,16]. Three months later, this process was repeated, but only for the anonymized mGRE images and at random. Using mGRE sequences, the presence of JHD was determined on the basis of a progressive signal drop to a signal void and the progressive accentuation of blooming artifacts on the successive images/echoes of the multi-echo sequence (Figure 1). The final decisions on the presence or absence of JHD for each individual ankle joint were made by the two MSK radiologists, by consensus, two months following the last reading and these were based on the total number of images for each patient; these readings served as reference. Moreover, the two MSK radiologists graded JHD as absent, mild, moderate, and severe [15] by consensus on the basis of the largest anterior–posterior dimension of the most hypointense area of the synovium (APS) in the fourth image (TE = 9.2 ms) of the multi-echo sequence; this image/echo was arbitrarily selected. Joint hemosiderin deposition was graded as mild (grade 1) for APS < 5 mm, moderate (grade 2) for APS > 5 mm and <10 mm, and severe (grade 3) for APS > 10 mm based on at least one pocket [16,28]. Since there is no standardized MR imaging protocol for measurements, we hypothesized that evaluation on the 4TE image/echo reflects a signal drop in the blooming artifact rather than real synovial thickness. At the same time, the other components of HA according to the International Prophylaxis Study Group (IPSG), i.e., synovial thickening, joint effusion, and osteochondral changes (OCC) [15], were also evaluated by the two MSK radiologists by consensus. In this study, synovial thickness was assessed using STIR images, which are less susceptible to paramagnetic properties of hemosiderin [27]. Since the focus of our study is exploring the capability of the mGRE sequence in the assessment of JHD, we considered the other components of HA as present or absent and did not grade them.

In order to obtain a quantitative measure of JHD, T2* relaxation times of the synovium were estimated in 12 randomly selected ankle joints with synovial thickness >3 mm. T2* relaxation maps were constructed using data obtained via the mGRE sequence by means of an inhouse-developed algorithm based on monoexponential fit and least-square methods. Readers A and B, by consensus, estimated the T2 values of ROIs, drawn at the thickest part of the synovium. This process was repeated blindly after three months by the same readers; however, there was a wide variation in the T2* values for each individual joint. Therefore, T2* mapping of the synovium with this technique was considered to be non-repeatable for the objective assessment of JHD.

### 2.4. Statistics

The degree of agreement of the readers’ judgements with the consensus reading was assessed primarily with the intraclass correlation coefficient (ICC) with a 95% Confidence Interval (CI), which shows the reliability of the judgements against the golden standard. Also reported are the sensitivity, specificity, accuracy, PPV (positive predictive value), and NPV (negative predictive value). The association between the JHD scores and the other imaging findings of HA (18), i.e., synovial thickening, effusion, OCCs, as well as the clinical score and the number of joint bleeds, was tested with the chi-square test. The association between the chronicity of joint bleeds (≥5 years, <5 years) and the presence or absence of JHD was tested with the chi-square test. Statistical significance was set at 0.05. Analyses were performed with the IBM SPSS Statistical package v.28.0.

## 3. Results

### 3.1. Detection of Joint Hemosiderin Deposition with mGRE Sequence: Inter-Reader Agreement

**Figure 2:**
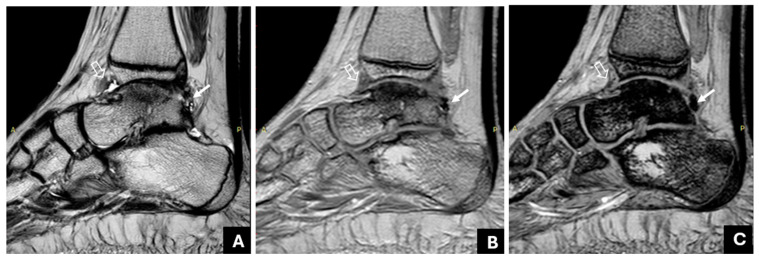
Right ankle joint of a 17-year-old patient with hemophilia and a history of 10 joint bleeds. (**A**): A sagittal T2W TSE shows mild thickening and hypointensity of the anterior and posterior synovium. An hypointense area is also seen adjacent to the anterior synovium (empty arrow). (**B**,**C**): The multi-echo GRE (mGRE) sequence does not show substantial signal reduction or blooming artifact in the anterior recess (empty arrow) in the successive image/echoes ((**B**): 1TE: 2.3, (**C**): 4TE: 9.2), suggesting synovial thickening without hemosiderin deposition. However, there is an increasing signal drop and blooming artifact in the posterior recess (arrow), implying the presence of hemosiderin. Osteochondral changes of the talus are also evident in all images.

The consensus reading assessed 21 joints (51.2%) with negative findings and 20 joints (48.8%) with positive findings for JHD. Table 1 shows the degree of agreement of the readers’ judgements with the consensus reading, assessed primarily with the ICC with a 95% CI. Multi-echo GRE could reveal subtle hemosiderin deposition, even for the least experienced reader C (Figure 1), whereas the determination of synovial thickening was possible without hemosiderin and JHD (Figure 2). For reader A only, the ICC from the conventional MR protocol was not significantly lower than the mGRE sequence.

### 3.2. The Association of Joint Hemosiderin Deposition with Synovial Thickening, Effusion, and Osteochondral Changes (OCC) 

**Figure 3:**
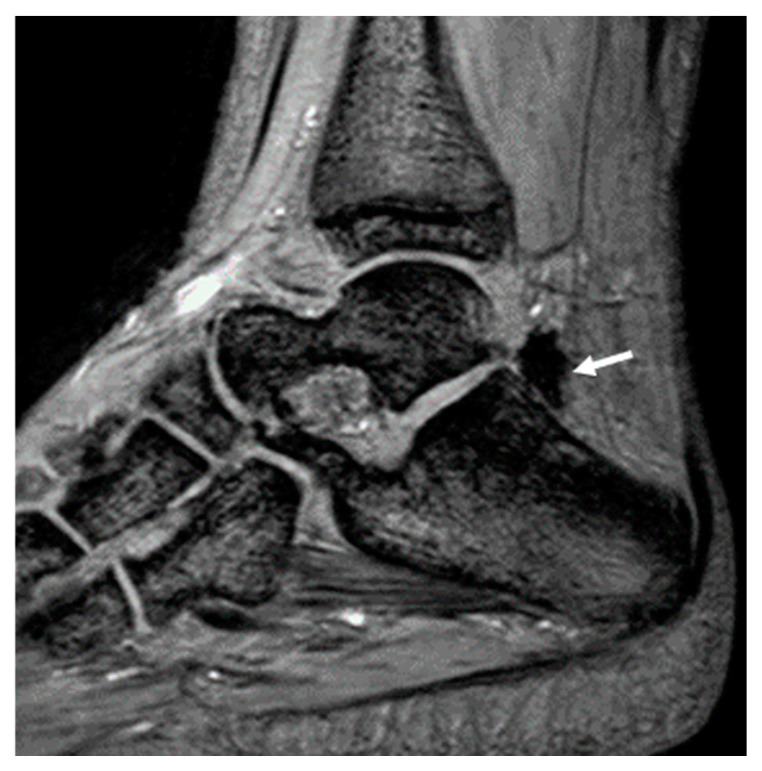
Left ankle joint of a 14-year-old patient with haemophilia and a history of nine joint bleeds. The fourth image (4TE: 9.2) of the mGRE sequence only shows significant hemosiderin deposition posteriorly (arrow).

**Figure 4:**
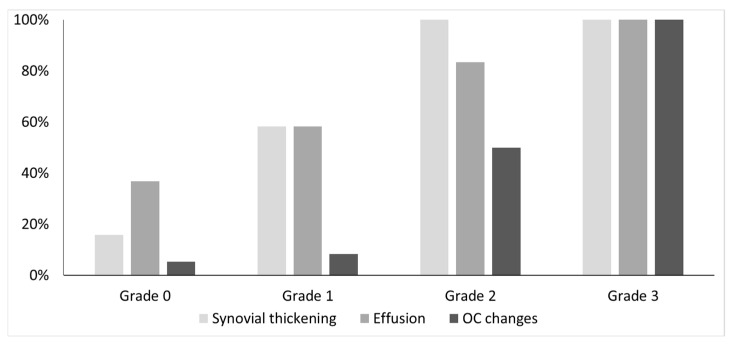
Association between grades of JHD (joint hemosiderin deposition) and synovial thickening, effusion and OC (osteochondral) changes. Both synovial thickening and OC changes were more common in patients with moderate or severe JHD. For patients without or mild JHD OC changes were uncommon but synovial thickening was not infrequent.

**Figure 5:**
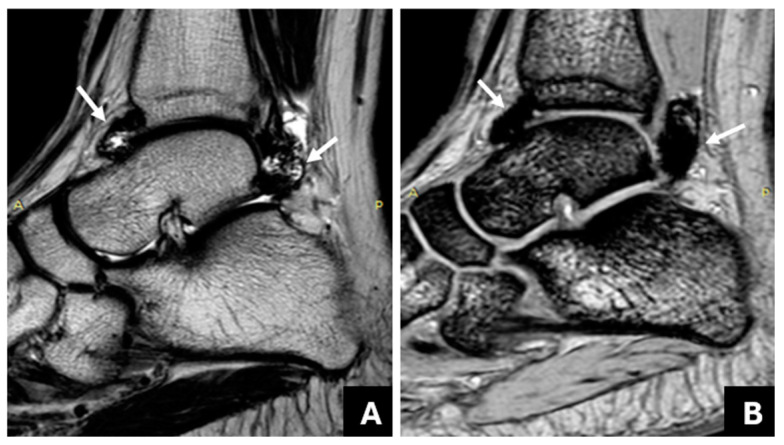
Left ankle joint of a 17-year-old patient with haemophilia and a history of eight joint bleeds. (**A**): A sagittal T2W TSE image demonstrates thickened and hypointense synovium with hyperplastic villi (arrows). (**B**): There was augmentation of the signal void and blooming artifact of the synovium (arrows) in successive sagittal images of the GRE sequence (4TE: 9.2). Synovial thickening was evaluated using the T2 image (**A**), where the blooming artifact was significantly less significant, whereas JHD was evaluated using mGRE images (**B**).

**Figure 6:**
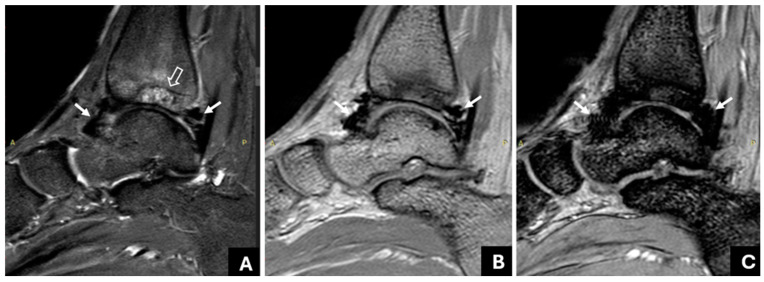
Right ankle of a 17-year-old male patient with haemophilia and a history of 10 joint bleeds. (**A**): A sagittal short-tau inversion recovery image of the right ankle denotes JHD with synovial thickening (arrow) and extensive osteochondral changes of the tibia, including bone marrow edema and subchondral cysts (empty arrow). (**B**,**C**): mGRE sagittal images ((**B**): 1TE:2.3; (**C**): 4TE: 9.2). Note that adjacent structures to the thickened and siderotic synovium are clear in the first image/echo (**B**) but obscured, due to augmented blooming artifact, in the later image/echo (4TE:9.2 (**C**)).

Table 2 presents patients’ demographics, clinical, and MR imaging data. The MR imaging findings presented in this table were decided based on the consensus reading of the two MSK radiologists. Joint hemosiderin deposition was detected in 22 of 41 joints, located at both the anterior and posterior recess in 15/22 joints (68%), whereas 7/22 joints (32%) denoted the involvement of only (or predominately) the posterior recess (Figure 2 and Figure 3). Isolated or predominant anterior involvement was not revealed in any patient. Figure 4 presents the associations between the grades of JHD and the presence of synovial thickening, effusion, and OCC. Synovial thickening was seen in 22 joints (Figure 5) and was associated with the presence and severity of JHD (*p* < 0.002). Four joints with mild JHD did not show synovial thickening, whereas four joints with synovial thickening did not present JHD. OCC were observed in 9 joints, all of them having JHD, and associated significantly with the degree of severity of JHD (*p* < 0.001) (Figure 6). Almost all patients with mild JHD (10/11) or without JHD (1/19) did not present OCC, whereas all patients (4/4) with severe JHD and 50% of patients with moderate JHD (3/6) presented OCC (*p* < 0.05). Effusion was detected in 23 joints and was also associated with JHD, albeit not so strongly (*p* < 0.04).

### 3.3. Association of Joint Hemosiderin Deposition with the Number and Chronicity of Joint Bleeds and Clinical Score

**Figure 7:**
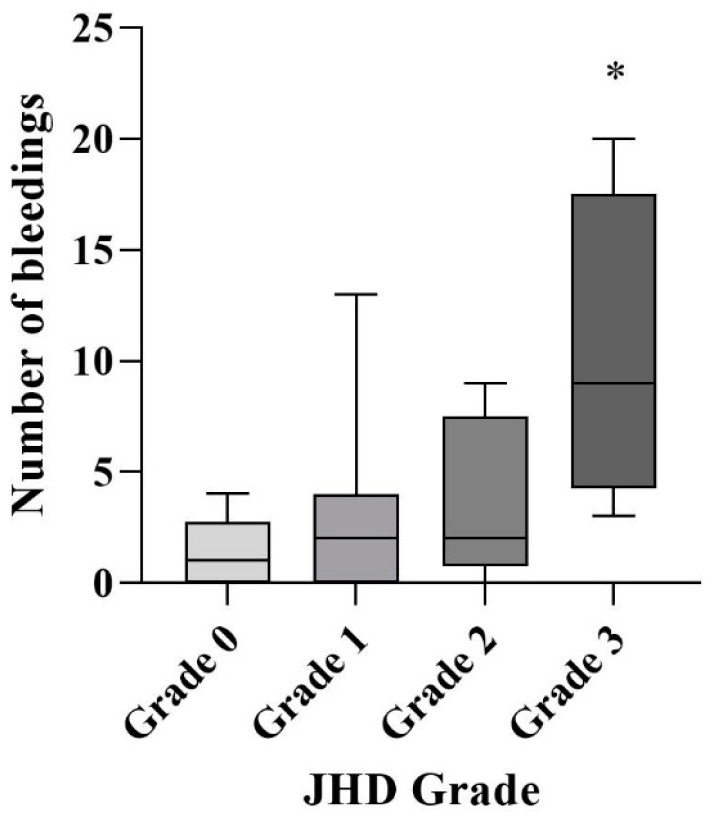
Number of JBs (joint bleeds) vs. grades of JHD (joint hemosiderin deposition). The number of previous JBs was significantly increased only for joints with severe JHD (grade 3) (*p* < 0.05). An asterisk (*) indicates statistical significance.

A past history of JBs had been registered in 32 ankle joints. Of the nine joints without any history of JBs, three exhibited mild or moderate JHD, which was attributed to subclinical bleeds (33%). The number of previous JBs was significantly increased only for joints with severe JHD, as shown in Figure 7, whereas mild or moderate JHD was not associated with the total number of JBs (*p* < 0.05). The clinical score (HJHS 2.1) was also associated with the grades of JHD (*p* < 0.05).

Subsequently, the ankle joints were categorized into two groups based on the time elapsed between the MR study and the last JB: (i) joints with the last reported JB less than five years prior (18 joints) and (ii) joints with the last reported JB five or more years before the current MR imaging (13 joints). Joints with a <5-year history of JBs showed significantly more frequently JHD (15/18 joints) compared to joints with a ≥5-year history of JBs (4/13 joints), *p* < 0.05.

## 4. Discussion

In the first part of the current study, we proposed the application of a multi-echo gradient-echo sequence (mGRE) for the assessment of intraarticular hemosiderin in the ankle joints of PWH. It could be easily inferred that the mGRE sequence yielded more satisfactory assessments than the conventional method for all the readers regarding sensitivity, specificity, and overall confidence. Only for reader A, who was the most experienced MSK radiologist in haemophilia imaging, was the ICC from the conventional MR protocol not significantly lower than that from the mGRE sequence. Of note, the performance of the less experienced reader C was significantly improved, as inferred by the high value, ICC = 0.976, obtained when using the mGRE sequence compared to the value, ICC = 0.481, derived when using the conventional protocol. This improvement was due to the increased susceptibility of the mGRE sequence to the paramagnetic prοperties of hemosiderin, resulting in a marked and gradual decrease in the signal intensity of the siderotic synovium along with the augmentation of the blooming artifact. These are apparent with a progressive increase in the echo time (Figure 1) and are pathognomonic of the presence of hemosiderin [20]. It should be noted that, using conventional MRI, both fibrosis and hemosiderin are hypointense [33], and it is not always possible to discriminate between them, especially in joints with mild JHD and for less experienced readers like the reader C. On the contrary, using an mGRE sequence, it was possible to discriminate between fibrotic/hypertrophied synovium without hemosiderin from hemosiderin deposition, as the former does not show the characteristic blooming artifact and progressive signal decrease in successive image/echoes (Figure 2).

It has been argued that single-echo GRE techniques impede the visualization of adjacent structures due to the strong blooming artifact [27]. However, using the mGRE sequence, the blooming artifact is less pronounced in the earlier image/echoes, allowing the visualization of adjacent structures, even in cases with severe JHD (Figure 6). Moreover, it is an easily applicable and available technique; our findings on a 3T MRI system could be extrapolated on 1.5 T MRI systems, too. To the best of our knowledge, interobserver variability between readers with different levels of experience has not been adequately addressed; in most previous studies, the interpretation of the MR studies of the haemophilic joints has been performed by radiologists with large amounts of experience in the imaging of haemophilic arthropathy [15,16,20,21]. Akuyz et al. refer to high interobserver agreement between readers with different levels of experience using susceptibility-weighted imaging (SWI) on 3 T [29], whereas, most recently, other research teams also reported using the increased sensitivity of SWI to identify JHD [34] or recent haemorrhages [35]. Few recent studies have used ultra-small TE sequences in the MR imaging of joints with haemophilia. In their work, Jang et al. constructed quantitative susceptibility maps of two knees and one ankle joint of PWH using ultrashort TE sequences (UTE-QSM) [36]; this sophisticated technique requires further validation to be introduced in clinical practice [36,37].

The mGRE sequences with the subsequent estimation of tissue T2* or R2* (R2* = 1/T2*) and the construction of T2* maps have been widely employed for the quantitative evaluation of liver and myocardial hemosiderosis in various pathologic conditions associated with iron overload, mostly referenced on 1.5 T and recently on 3 T MRI systems [38]. We attempted to construct T2* maps of the synovium based on measurements obtained by the mGRE sequence, but quantitative results presented large interobserver variability; the irregular shape and inhomogeneous composition of the synovium included in a voxel, unlike the more homogenous structure of liver or myocardium, and the increased signal decay rate (ultrasmall T2* relaxation time) [35] may account for this.

It has been demonstrated that ulrasound, when performed by pediatric radiologists, is a reliable tool for detecting and quantifying HA in children compared to conventional MRI [16,39]. However, Plut et al. found that correlations between ankle joint assessment were the lowest for all components of HA, as US struggles to visualize the deep parts of the ankle joints [39]. Prasetyo et al. documented even weaker correlations between US and a single-echo GRE sequence in ankles with HA, since US often could not discriminate between JHD and synovial thickening or fat pads [40], in alignment with findings by von Dygalski et al. [26]. We did not compare US with the mGRE sequence, but even bigger disagreement can be expected due to enhanced capabilities of the mGRE to visualize confidently JHD and discriminate from adjacent soft tissues.

In the second part of the study, we investigated the association between JHD with the other components of HA, i.e., synovial thickening, effusion and OCC, which—to our knowledge—have not been adequately explored [8,17,28]. We found that severe JHD was closely associated with OCC, whereas mild or a lack of JHD rarely coexisted with OCC. Blood degradation products, due to multiple and repetitive joint bleeds, are embedded within the synovial tissue, inciting synovial proliferation, vascular remodeling, and the release of inflammatory factors locally, while, at the same time, having a direct toxic effect on the joint cartilage, leading progressively to HA [2,3] with severe OCC; therefore the prompt identification of hemosiderin and administration of prophylactic treatment may obviate the deleterious effects of HA. Recently, Foppen et al. reported that synovial hyperplasia and JHD were strong predictive factors for the rebleeding of the joint within the next five years, whereas early MR changes predicted the development of HA [19]; the prompt identification of JHD with the mGRE sequence can alter patient monitoring and intensify treatment according to individualized patients’ physical exercise and real needs [5]. The mGRE sequence could be further employed for the knees and elbows of patients with haemophilia on different MR imagers, using both 3 T and 1.5 T. In our study, a general radiologist without experience of HA imaging was able to identify confidently even mild JHD by means of the mGRE sequence at similar rates to the most experienced reader; all being the same, clinical hematologists might perceive the presence and severity of JHD on mGRE more easily and confidently for both the initial assessment and follow up of PWH.

JHD and synovial hypertrophy usually coexist and are often used interchangeably in the literature. Herein, we use the more general term “thickening” instead of “hypertrophy” to include both the hypertrophy of the synovium with apparent hypertrophied villi and the non-specific synovial thickening that may be due either to hypertrophy or other causes, such as fibrosis. Unlike previous studies on MR imaging of HA [8,9,10,11,12,15,17,18,19,28], in this cohort, we evaluated synovial hypertrophy/thickening and JHD with different sequences: synovial thickening was evaluated by means of the short-tau inversion recovery (STIR) or a T2 sequence, which are less susceptible to the paramagnetic properties of hemosiderin, in order to avoid the overestimation of synovial thickness due to blooming artifacts [27], whereas JHD was assessed with an mGRE sequence, considering the progressive signal drop and blooming artifact as typical findings for JHD. We did not administer intravenous gadolinium to evaluate synovial thickness, since it is not considered necessary in HA, unlike in rheumatoid arthritis [41]. We addressed, also, the distribution of hemosiderin within the synovium; it usually affects both the anterior and posterior recess, although involvement being limited to the posterior recess is not uncommon (Figure 3). The clinical significance of this finding for the possible development of posterior impingement should be further explored.

In this study, MR imaging revealed JHD in 33% of joints without a history of prior bleeding, presumably due to subclinical bleeds; the higher frequency of subclinical bleeds revealed in this series compared with previous studies even of the same group is probably due to the more sensitive and accurate detection of hemosiderin using the mGRE sequence, given that patients of this cohort receive early and regular prophylactic treatment [5,6]. On the other hand, JHD was more frequently seen in recent bleeds and was lacking in 64% of joints with a history of JBs that had occurred at least 5 years before the current MR study, in agreement with Uijl et al. and Zuagenmaker et al. [10,28]. Pergantou et al. reported that mild or moderate JHD could be a reversible process that might not leave residual hemosiderin if prompt and adequate prophylactic treatment is administered [17]. Both the HJHS2.1 score and the number of JBs were associated with JHD in the current series; previous studies report contradictory results for these associations due to the variable experience of clinical examiners [42] and also the variability of MR imaging techniques and evaluation scales [8,9,10,11,12,17,18,28,29]. The standardization of MR imaging protocols is mandatory to assure comparability between studies and provide the maximum accuracy within the shortest possible examination time [21,42].

We evaluated only one joint type, the ankle joint, and this is a limitation of our study. Other limitations include the small number of ankle joints and the fact we have not compared the performance of the currently used mGRE sequence to the more commonly used single-echo GRE-based sequences, the most recently employed SWI method, or ultrasound. The lower age limit in our study was nine years; younger patients may need sedation and this a drawback of MRI in children in general. We also lack confirmation of MRI findings with histopathology, as ankle surgeries in pediatric PWH are rarely performed nowadays, due to early prophylaxis, whereas biopsies of the synovium are unethical to perform.

In conclusion, we applied a multi-echo GRE sequence that provided sensitive and specific identification of JHD, even when employed by less experienced radiologists. MRI can directly visualize the amount of JHD, while haemarthroses often may not show JHD on MRI when they are past being mild. The severity of JHD was found to be associated with the other components of HA.

## Figures and Tables

**Table 1:** Agreement of readers’ judgments with consensus reading.

Method	Radiologist	Sensitivity	Specificity	PPV	NPV	ICC
Conventional	A ^1^	85.0% (64.0–94.8%)	95.2% (77.3–99.2%)	94.4% (74.2–99.0%)	87.0% (67.9–95.5%)	0.894 (0.802–0.943)
	B ^2^	80.0 (58.4–91.3%)	76.2% (54.9–89.4%)	76.2% (54.9–89.4%)	80.0% (58.4–91.3%)	0.724 (0.480–0.853)
	C ^3^	50.0% (29.9–70.1%)	81.0% (60.0–92.3%)	71.4% (45.4–88.3%)	63.0% (44.2–78.5%)	0.481 (0.049–0.720)
T2*	A ^1^	90.0% (69.9–97.2%)	100% (84.5–100%)	100% (82.4–100%)	91.3% (73.2–97.6%)	0.950 (0.906–0.973)
	B ^2^	95.0% (76.4–99.1%)	95.2% (77.3–99.2%)	95.% (76.4–99.1%)	95.2% (77.3–99.2%)	0.950 (0.906–0.973)
	C ^3^	95.0% (76.4–99.1%)	100% (84.5–100%)	100% (83.2–100%)	95.5% (78.2–99.2%)	0.976 (0.954–0.987)

^1^: 18 years of experience in MSK imaging and 13 years of experience in haemophilic arthropathy (HA); ^2^: 9 years of experience in MSK imaging and 4 years of experience in HA; ^3^: general radiologist, non-specialized in MSK imaging or HA; PPV: positive predictive value; NPV: negative predictive value; ICC: Interclass Correlation Coefficient. In parentheses, we see the 95% Confidence Intervals.

**Table 2:** Patients’ demographics, clinical, and MR imaging data.

Pt, No	Age	Rt/Lt Ankle	JBs, n	Years from Last JB	Clinical Score	JHD, Grades	Synovial Thickening	Effusion	OC Changes
1	17	Rt	13	1	1	1	+	+	+
		Lt	1	7	2	0	−	+	−
2	9	Lt	4	1	1	1	−	+	−
3	13	Rt	0	0	0	0	−	−	−
		Lt	4	9	1	0	+	+	−
4	12	Rt	0	0	0	1	+	+	−
		Lt	1	9	0	0	−	−	−
5	12	Rt	2	0.5	0	0	+	−	−
		Lt	4	1	0	0	+	−	−
6	16	Rt	4	0	0	1	−	−	−
		Lt	2	2	0	1	+	+	−
7	16	Rt	0	0	0	0	−	−	−
		Lt	1	5	0	1	−	−	−
8	14	Rt	4	4	0	1	+	+	−
		Lt	0	0	0	0	−	−	−
9	16	Rt	2	7	1	1	+	+	−
		Lt	3	2	1	3	+	+	+
10	14	Rt	3	1	1	1	+	−	−
		Lt	1	4	0	2	+	+	−
11	15	Rt	7	6	3	2	+	+	+
		Lt	9	3	0	2	+	−	+
12	9	Rt	0	0	2	0	+	−	+
		Lt	1	2	2	1	+	−	−
13	16	Rt	2	8	0	2	+	+	−
		Lt	20	1	1	3	+	+	+
14	14	Rt	0	0	2	2	+	+	−
		Lt	2	1	2	2	+	+	+
15	17	Rt	0	0	0	0	−	+	−
		Lt	4	12	0	0	−	+	−
16	17	Rt	0	0	0	0	−	−	−
		Lt	2	6	0	0	−	+	−
17	17	Rt	8	0.5	0	3	+	+	+
		Lt	2	9	0	1	−	−	−
18	17	Rt	1	5	2	0	−	+	−
		Lt	2	1	2	1	−	+	−
19	17	Rt	10	0.5	1	3	+	+	+
		Lt	0	0	0	0	−	−	−
20	15	Rt	1	6	0	0	−	−	−
		Lt	1	0.5	0	0	−	+	−
21	16	Rt	2	5	0	0	−	−	−
		Lt	4	11	0	0	+	−	−

“+” for positive and “−” for negative on MRI. Abbreviations: Pt, No: serial number of each patient; Rt/Lt: right/left; Years from Last JB: time elapsed (in years) from the last joint bleed; Clinical Score: clinical score according to the HJHS 2.1 clinical scoring system (29); HJD: hemosiderin joint deposition; OC Changes: osteochondral changes.

## Data Availability

The raw data supporting the conclusions of this article will be made available by the authors on request.
